# Cybersecurity governance in the healthcare sector during digital transformation: an integrated model and hybrid analytical approach

**DOI:** 10.3389/fpubh.2025.1703689

**Published:** 2025-11-19

**Authors:** Amnah Alharbi, Ali Alkhalifah

**Affiliations:** Department of Information Technology, College of Computer, Qassim University, Buraydah, Saudi Arabia

**Keywords:** cybersecurity, digital transformation, healthcare, protection motivation theory, general deterrence theory, privacy

## Abstract

**Introduction:**

Digital transformation is increasingly relied upon in the healthcare sector, enhancing service efficiency but posing security challenges related to privacy and trust. With the increasing use of digital technologies, cybersecurity issues are becoming more critical, especially given the risks of breaches and data leaks. Therefore, understanding the impact of security factors on employee security behavior during digital transformation is critical.

**Methods:**

Based on general deterrence theory and protection motivation theory, this study developed a research framework for examining digital transformation factors, such as complexity (the interconnectedness of diverse digital health systems) and exploitability (the potential for vulnerabilities in those systems to be leveraged by attackers), and cybersecurity-related factors, such as privacy, trust, and awareness, and to understand how they influence employee behavior in healthcare. Data were collected from 252 healthcare workers in Saudi Arabia and analyzed using structural equation modeling and artificial neural networks.

**Results:**

The results showed that trust, exploitability, awareness, and certainty of punishment significantly impact security behavior during digital transformation. Privacy concerns and complexity were also found to significantly influence threat assessment and response evaluation. However, consistent with some studies in managed security environments, perceived vulnerability, perceived threat, and self-efficacy had no impact on security behavior. Finally, the study presents its theoretical and applied contributions and recommendations for future research.

## 1 Introduction

Digital transformation is one of the most significant changes the world has witnessed recently, as organizations rely on digital technologies such as cloud computing, big data, artificial intelligence, and smart communications to improve their operations and deliver more efficient and high-quality services (1).

In the healthcare sector in particular, digital transformation has become an essential element for providing safe and rapid healthcare services, such as electronic health records, telemedicine, and data analytics to support medical decision-making (2).

This transformation contributes to improving the quality of care, reducing errors, and increasing patient satisfaction. Also, it represents an important step toward achieving a future vision based on innovation and efficiency. Research conducted by Deloitte (3) in 2025 indicates that nearly 90% of C-suite executives expect the use of digital technologies to expand in healthcare organizations. In this regard, cybersecurity has become one of the most significant challenges facing healthcare organizations during the digital transformation phases. While digital transformation contributes to improving efficiency and facilitating access to services, it also increases the chances of being exposed to cyberattacks and security breaches (4). The healthcare sector experienced the most expensive data breaches for the 13th consecutive year, with an average cost of $10.93 million, up from $10.10 million in 2022, according to IBM’s 2023 Cost of a Data Breach Report (5). Moreover, Data breaches in the healthcare industry significantly increased in 2024. Over 45 million health records were compromised in 387 significant data breaches that were reported in the first half of the year (6).

As 2025 begins, cyber threats to healthcare organizations are constantly evolving, with artificial intelligence (AI)-powered attacks becoming a growing threat alongside the continued spread of ransomware (7). Cybersecurity experts report that these threats continue to pose significant challenges to healthcare organizations in securing their data and digital infrastructure (7), such as privacy breaches, unauthorized access to services and data, and a lack of awareness about the use of these systems. This makes cybersecurity a top priority to ensure data protection, patient safety, and the success of the digital transformation process (4). Therefore, this study focuses on the factors influencing digital transformation adoption from a cybersecurity perspective.

Despite years of research efforts to address the challenges and breaches related to digital technologies (8–10), research gaps still exist that require further study to shed light on the cybersecurity needs arising from the digitization of services, especially in the healthcare sector, which is a target for attackers due to the sensitivity of its data, reinforcing the need for this focus (11). Several studies (12, 13) indicate that most breaches stem from the human factor. Although the human factor represents a major vulnerability in cybersecurity, it has been the subject of only a few studies to date. Negligence or a lack of secure behavior by employees can lead to serious breaches, compromising patient privacy and data (13, 14).

Therefore, exploring the factors influencing employee commitment to security practices is critical, especially in an environment where cyberthreats are increasingly complex. Only limited research has been conducted to address employee behavior in the context of cybersecurity during the digital transformation of the healthcare sector, particularly in Saudi Arabia (15). This calls for further research to understand these aspects and enhance effective security policies and protection strategies for Saudi healthcare organizations.

This highlights the need to explore employee behavior regarding cybersecurity and provide solutions that enhance the security of digital systems, as well as assist in developing strategies to protect Saudi healthcare organizations. Accordingly, this study aims to explore factors that influence cybersecurity behaviors among healthcare employees during digital transformation, develop a research model that integrates two behavioral theories general deterrence theory (GDT) and protection motivation theory (PMT) to examine how deterrents, perceived threats, and coping mechanisms influence security behaviors and investigate the relationships between digital transformation factors (e.g., complexity and exploitability) and human-related factors (e.g., awareness, privacy, and trust) in shaping employees’ cybersecurity behavior. The research questions explored in this study are as follows:

RQ1: What are the key factors that affect cybersecurity during digital transformation in the healthcare sector?

RQ2: To what extent do these factors influence the cybersecurity behaviors of healthcare employees during digital transformation?

## 2 Literature review and theoretical background

### 2.1 Digital transformation: reshaping services in healthcare

Regulation (EU) 2021/694^1^ defines digital transformation as the use of digital technologies to drive service changes and business across sectors such as finance, telecommunications, and healthcare (16). A study (17) describes it as a process that enables major organizational improvements—like enhanced customer experiences, streamlined operations, and new business models—through technologies such as mobile tools, analytics, embedded devices and social media.

In healthcare, digital transformation involves using technologies to enhance service delivery, improve problem solving, and achieve better patient outcomes. Tools such as telemedicine platforms and electronic health records improve emergency response, while real-time analytics and machine learning help Identify healthcare providers can identify critical health issues quickly. As healthcare evolves, digital transformation emphasizes patient engagement, viewing patients as active participants who seek personalized, convenient, and immediate care. This shift promises higher productivity, greater efficiency, and lower infrastructure costs. However, studies (18, 19) remark that current literature on digital transformation in healthcare remains limited, as it often overlooks patient-defined value and fails to integrate new value models with traditional healthcare operations.

The Kingdom of Saudi Arabia (KSA) actively supports digital transformation, establishing the Digital Government Authority (DGA) and ranking first in 2022 for electronic and mobile government service maturity in the UN ESCWA Index, which includes healthcare services. Digital health is a key component of the Ministry of Health’s Vision Realization Office programs, aiming to enhance public health through value-based care (20). For example, a leading national initiative is the *Sehhaty* app, launched by the Ministry of Health to provide citizens and residents with easy access to services such as appointment booking and remote consultations. AI-powered diagnostic tools have also been implemented in several hospitals to support early detection of chronic diseases like cancer and diabetes. Additionally, the National Unified Medical Record (NUMR) initiative improves patient data management through a secure, centralized system for information exchange among healthcare providers (21).

The adoption of digital systems has widened the cyberattack surface, creating complex security challenges (22). Sensitive healthcare data and services are highly vulnerable to breaches, putting patient privacy and safety at risk. Global reports (5, 6) show that healthcare is among the most targeted sectors due to the high value of its data. Studies (23–25) highlight rising cyberattacks that threaten not only patient privacy but also safety, operations, and financial stability. As these threats grow, strong cybersecurity measures and polices have become essential to protect healthcare infrastructure. Although digital transformation improves care quality and efficiency, it also demands robust cybersecurity and prevention efforts.

### 2.2 Role of cybersecurity in healthcare systems

Cybersecurity represents one of the fundamental pillars for ensuring the success of digital transformation (26). The evolution of cybersecurity can be traced back to the early days of computing when security concerns were relatively rudimentary. In the 1970s, the emergence of computer networks marked a shift in the threat landscape, necessitating the development of measures to protect sensitive data. The concept of firewalls and encryption began gaining prominence in the 1980s as Internet usage became more prevalent. The 1990s saw a surge in cyberattacks, prompting the establishment of dedicated cybersecurity teams. The evolution of cybersecurity from simple virus production to the complex advanced persistent threats (APTs) of today is depicted in the history of the field (27). These APTs, with their stealthy and continuous nature, have become major challenges, highlighting the need for ongoing advancements in cybersecurity. The primary goal of cybersecurity is the protection of digital assets and information systems, ensuring protection against unauthorized access, theft, and damage while upholding the confidentiality, integrity, and availability (CIA) of data (28). Cybersecurity faces a significant challenge due to the continually evolving landscape of cyber threats. Attackers employ diverse techniques, such as malware, phishing, social engineering, and ransomware, to exploit vulnerabilities within computer networks and systems. The CIA principles form the cornerstone of information security. Cyberattacks often aim to compromise one or more of these principles. For instance, ransomware attacks, a prevalent modern threat, exploit vulnerabilities to encrypt data, thereby compromising its integrity and availability (13).

The historical development of information security, derived from these CIA principles, provides a foundation for understanding the challenges posed by cyber threats and the necessity for proactive measures to uphold this security triad of principles. As the historical narrative of cybersecurity unfolds, embracing the CIA triad becomes essential in mitigating the impact of evolving cyber threats. In the realm of healthcare, where the digital transformation of services is underway, adherence to these principles, fortified by international security standards, serves as a robust defense against cyber adversaries seeking to compromise critical health data (13, 27). Interestingly, while digital technologies may introduce new risks to the healthcare environment, they also offer significant opportunities for developing advanced solutions that enhance data security and system integrity, if implemented thoughtfully and securely.

Previous literature (10, 29, 30) has extensively discussed the technical solutions developed in the field of cybersecurity to enhance the protection of digital infrastructure in the healthcare sector. These solutions have included multiple technologies such as advanced encryption (31), blockchain implementation (8), and the development of identity and access management (IAM) systems (32), all of which aim to limit unauthorized access and ensure the confidentiality and integrity of sensitive health data. The use of artificial intelligence and machine learning to analyze suspicious behavior and detect attacks early has been widely applied in healthcare settings. For example, Ghourabi (33) developed a hybrid system based on LightGBM and a Transformer-based model to target malware and intrusion attacks on medical devices and data servers. The system achieved up to 99% accuracy thanks to a variety of training datasets, including attacks from Internet of Things (IoT) and Internet of Medical Things (IoMT) environments.

In addition, the study (34) proposed an intelligent intrusion detection system that targets the IEC 60870-5-104 protocol, commonly used in medical industrial systems. The system relied on the integration of machine learning techniques with software-defined networking (SDN) and was able to analyze both network flows and packet content to automatically detect complex attacks. Furthermore, Hady et al. (35) demonstrated that integrating network metrics with patient biometric data into intrusion detection systems enhances the system’s accuracy and increases its ability to predict attacks. These findings confirm that modern technologies provide advanced and effective solutions for monitoring the growing cyber threats in the healthcare sector, as they are characterized by their ability to adapt to new types of attacks and analyze the vast amount of health data in real time. Therefore, integrating these technologies into the digital infrastructure of healthcare institutions is a fundamental step toward enhancing cybersecurity and ensuring the continuity of medical services without interruption or risk to patients (8).

Despite the importance of these technological innovations and their pivotal role in enhancing security, most studies have focused primarily on technical solutions (36–38), with relative neglect of behavioral aspects and the study of individual and technical factors related to users, such as security awareness, preventive behaviors, trust, and perception (39). Multiple studies (2, 13, 40) have shown that the human factor remains one of the most prominent weaknesses in the cybersecurity chain, as employee negligence or lack of awareness can lead to serious breaches. Therefore, there is a need to expand the scope of research to include a deeper understanding of the behavioral factors associated with digital transformation that influence employee commitment to security practices, especially in the healthcare environment characterized by technical complexity and high workload.

### 2.3 Governance: strategic planning for compliance and security

In healthcare, governance is not simply about making rules; it is about careful planning. Strategic governance includes the implementation of robust access controls, continuous monitoring systems, and adherence to dynamic cybersecurity standards. Internationally recognized frameworks, such as ISO 27001, ensure that key security controls, such as access controls and monitoring, are in place. Standards of the United States (US) National Institute of Standards and Technology (NIST) provide a structured approach to managing information security and fortify the governance framework, ensuring resilience against evolving cyber threats while addressing risk in the healthcare landscape (41). This technical planning establishes a strong foundation for secure healthcare operations in accordance with global best practices. The Kingdom of Saudi Arabia (KSA) has paid significant attention to developing cybersecurity strategies and strengthening governance in this field. Several measures and initiatives have been undertaken to improve cybersecurity governance, including the creation of the document Essential Cybersecurity Controls (ECC-1:2018), published by the National Cybersecurity Authority (NCA) in 2018 (20). This document specifies a set of minimum cybersecurity controls that institutions in the country should have implemented to protect themselves from cyberattacks. The agreement applies to all Saudi Arabian organizations, including healthcare organizations.

The current study plays a pivotal role in enhancing cybersecurity governance by providing a more profound understanding of employees’ readiness to confront cyber risks and their awareness of the importance of compliance with controls. Therefore, this paper helps decision-makers design flexible governance policies based on realistic foundations that align with organizational culture and human factors.

### 2.4 Theoretical background

#### 2.4.1 Protection motivation theory (PMT)

Protection motivation theory (PMT) posits that individuals take preventive action when they perceive a serious threat and feel they can act effectively to mitigate that threat. The theory proposed by Rogers in 1975 and originally developed to understand preventive behavior in the healthcare field (42, 43), has expanded to include other areas, such as cybersecurity behavior in the digital world (44, 45). With the increasing reliance on digital technology in the healthcare sector, cyber threats have become more prevalent, posing new challenges for individuals and hospitals in maintaining data security (46). Protection motivation theory (PMT) is an important framework for understanding how individuals respond to security threats and take action to protect themselves from risks (15). By analyzing components of the theory, such as threat severity perception, vulnerability, response efficacy, and self-efficacy, how individuals deal with digital threats and make decisions to protect their data and systems (47) can be explained. For example, individuals are increasingly aware of cyber threats such as cyber-attacks, data theft, and cyber fraud. This awareness drives users and organizations to take preventive action to protect their data (45). This study’s contribution is to use PMT to help explain how employees respond to risks and threats, as well as to investigate whether these threats are related to digital transformation, such as complexity, or to cybersecurity, such as privacy and awareness. Many studies (45, 48, 49) have proven the effectiveness of this theory in the cybersecurity and healthcare sectors. Accordingly, PMT was chosen as a powerful framework for understanding employee responses to different threats and exploring the impact of these threats on employee cybersecurity behavior.

#### 2.4.2 General deterrence theory (GDT)

General deterrence theory (GDT) is a legal and social theory that aims to deter individuals from committing crimes or illegal behavior through the threat of sanctions (50). The theory is based on the idea that individuals make their decisions based on their analysis of potential costs and rewards. If the potential punishment for illegal behavior is sufficiently severe and guaranteed, individuals will choose to refrain from that behavior (4). General deterrence theory (GDT) comprises three main factors: severity of punishment, certainty of punishment, and speed (promptness) of punishment (51).

With rapid digital transformation and the reliance of healthcare institutions on technology to store and process sensitive patient data, cyber threats and cyber-attacks targeting this sector have increased, making it necessary to implement effective deterrence mechanisms to prevent cybercrimes (52). From the GDT perspective, severe sanctions can be applied to individuals or entities that violate the security of health data, whether through cyber-attacks or theft of patient information (15), for example, imposing large fines and criminal penalties on organizations that fail to adequately protect patient data and violate patient privacy. These penalties increase the obligation of healthcare organizations to take strict measures to ensure data security, such as encrypting information and using advanced security protocols.

One of the reasons this study chose GDT is that only limited research was available on the impact of punishment on employee cybersecurity behavior in the healthcare context (15, 51, 53). Furthermore, previous studies (15, 54) have shown that the presence of confirmed punishment is important for employee compliance with security policies. Therefore, this theory also provides a strong basis for understanding how punishment affects employee behavior during digital transformation (53). Based on the above points, the current study proposed a model to examine the impact of the certainty of punishment on the digital transformation factor (exploitability) and on the behavior factor (trust).

## 3 Model development and hypotheses

**Figure 1:**
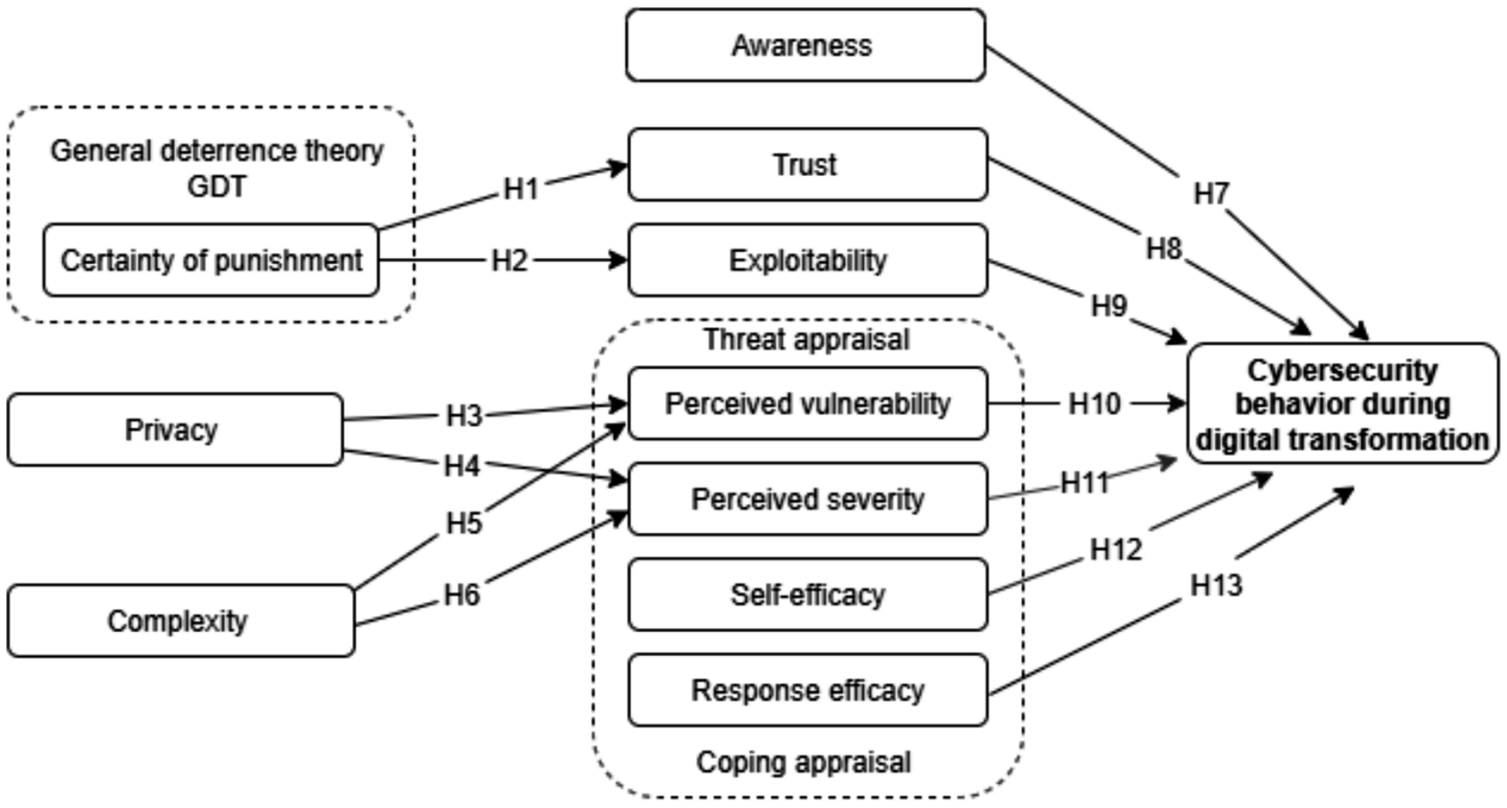
Research model.

Figure 1 illustrates the research model for this study which aims to explore cybersecurity behavior during digital transformation among healthcare employees, based on two main theories, the protection motivation theory (PMT) and the general deterrence theory (GDT), as previously discussed. The model is based on constructs derived from PMT, such as perceived severity, perceived vulnerability, self-efficacy, and response efficacy, in addition to assured punishment from the GDT to evaluate the impact of punishment. The model includes factors related to digital transformation, such as complexity and exploitability, in addition to human factors, such as trust, privacy, and awareness. This integrated framework aims to understand the interconnected impact of these factors on employees’ cybersecurity behavior.

Based on protection motivation theory (PMT), general deterrence theory (GDT), and factors related to security and digital transformation, the current study developed and applied an integrated model with 13 research hypotheses between the constructs to understand the cybersecurity behavior of employees during digital transformation of the healthcare sector, as shown in Figure 1. Table 1 presents the definitions of the constructs with the hypotheses discussed in detail in the following subsections.

**Table 1:** Definitions of constructs.

Construct	Definition	References
Perceived vulnerability	The likelihood that an individual will become the target of an unexpected event, for example, data breach, identity theft, or cyber-attack	(56)
Perceived severity	An individual’s perception of the consequences of potential threats	(47)
Self-efficacy	The extent to which individuals are confident in their ability to take preventive actions to protect themselves from threats	(56)
Response efficacy	The extent to which individuals believe that the preventive measures they are taking will be effective in protecting themselves from threats	(60)
Privacy	Protecting personal data and sensitive information from unauthorized access or manipulation	(55)
Awareness	Users’ understanding of the severity and potential impact of cyber threats, and their knowledge of the measures needed to avoid these threats	(44)
Complexity	The increase in digital systems and devices and their interconnections with each other	(57)
Certainty of punishment	Indicates that the certainty of punishment is a major factor in deterring individuals from committing crimes and violations	(51)
Trust	The level of confidence and belief that employees have in the ability of a system to perform its functions efficiently and reliably	(63)
Exploitability	The process of exploiting vulnerabilities or weaknesses in a digital system, network, or software to achieve illegal goals, such as stealing data or destroying systems	(68)

### 3.1 Certainty of punishment

The certainty of punishment factor is derived from the general deterrence theory (GDT), which indicates that the certainty of punishment is a major factor in deterring individuals from committing crimes and violations (51). In the digital environment, attackers can exploit many loopholes to achieve their malicious activities, such as violating privacy (15). Therefore, the certainty of punishment may be considered a deterrent and reduce their activity. Moreover, one study (54) confirmed that the level of prevention increases by efforts to avoid punishment. Based on the above, it is likely that the certainty of punishment will reduce the misuse of digital systems. Therefore, the following hypothesis is developed:

*H1*: Certainty of punishment has a positive effect on reducing exploitability.

Based on GDT, Kuo et al. (51) found a positive relationship between the certainty of punishment and the compliance with security policies of employees working in the healthcare sector. Their sense of the certainty of punishment enhanced their confidence in digital systems and increased their commitment to digital security policies. Based on Kuo et al.’s (12) previous study, the following hypothesis is posited:

*H2*: Certainty of punishment has a positive effect on trust.

### 3.2 Privacy

Privacy means protecting personal data and sensitive information from unauthorized access or manipulation (55). Individuals are increasingly exposed to privacy threats in the digital age, especially in the healthcare sector where health records and digital systems contain highly sensitive data (2). This exposure leads to concerns about the potential for privacy violations by unauthorized parties, such as hackers or even companies that collect data for commercial purposes (55). Protection motivation theory (PMT) views the loss of privacy as a threat that motivates individuals to protect their data. The study (56) examined the impact of threat severity, perceived vulnerability, response efficacy, and self-efficacy in relation to privacy. The study (56) found that individuals concerned about their privacy may feel more vulnerable to privacy threats, may perceive the consequences of privacy violations as more severe, and are more likely to adopt protective behavior to protect their privacy in digital environments, for example, by using encryption technologies and periodically reviewing privacy settings. Based on this previous study, the following hypotheses are proposed:

*H3*: Privacy positively affects perceived vulnerability.

*H4*: Privacy positively affects perceived severity.

### 3.3 Complexity

Complexity refers to the increase in the number of digital systems and devices and their interconnections with each other (57). With the development of digital technologies in the healthcare sector, systems are becoming more intertwined and interconnected, leading to increased complexity. This complexity is one of the main factors responsible for cybersecurity issues, as it hinders the safe adoption of information systems in this vital sector (58). The more complex the systems are, the more difficult they are to manage and make secure; thus, they are more vulnerable to cyber-attacks (58). Therefore, complexity and security are interconnected; when the system becomes more complex, it becomes less secure, adding a threat to the healthcare environment (59). The current study thus posits the following hypotheses:

*H5*: Complexity positively affects the perception of vulnerability.

*H6*: Complexity positively affects the perception of threat severity.

### 3.4 Awareness

Awareness refers to users’ understanding of the severity and potential impact of cyber threats, and their knowledge of the measures needed to avoid these threats (44). Studies (60, 61) have shown that the more aware an individual is of the existence and severity of cyber threats, the more likely they are to perceive the severity and potential impact of those threats. Moreover, awareness of the risks of digital systems in healthcare increases the perception of threats and their severity (62). Therefore, the current study hypothesizes that:

*H7*: Awareness has a positive effect on cybersecurity behavior during digital transformation.

### 3.5 Trust

Trust in digital technologies refers to the levels of confidence and belief that employees have in the ability of a system to efficiently and reliably perform its functions (63) Many studies (47, 64, 65) have found that trust in technologies is closely linked to cybersecurity behavior. When users trust the systems and technologies on which they rely, they are more willing to comply with required security practices (66). They feel that the data they share are well protected, which reduces concerns about privacy violations or exposure to cyber-attacks. Consequently, they adhere to the security policies imposed by these systems. Based on the above, the current study hypothesizes that:

*H8*: Trust has a positive impact on cybersecurity behavior during digital transformation.

### 3.6 Exploitability

During digital transformation, the healthcare sector has adopted many digital devices to enhance patient care and improve their daily lives (67). However, the presence of bugs and vulnerabilities or the use of outdated systems poses a significant risk of exploitation, and to the security, of these devices. Exploitation refers to the process of exploiting vulnerabilities or weaknesses in a digital system, network, or software to achieve illegal goals, such as stealing data or destroying systems (68). Exploitation significantly impacts digital transformation security in hospitals by increasing cyber risks, disrupting operations, increasing response and recovery costs, and damaging hospitals’ reputations (22). Many studies (12, 22, 68) have focused on the exploitability of vulnerabilities at the level of technical systems and infrastructure; however, exploitability at the employee level has not been studied. One study (69) stated that the weakest link in cybersecurity is human error, with employees in healthcare sectors creating ongoing security vulnerabilities, such as mismanagement of credentials, exposure of sensitive information, and improper authentication. However, if employees feel these vulnerabilities may be exploitable in digital systems, they will be more careful to adopt good cybersecurity behavior. Based on the above, the following hypothesis is posited:

*H9*: Exploitability has a positive impact on cybersecurity behavior during digital transformation.

### 3.7 Perceived vulnerability

Perceived vulnerability refers to the likelihood that an individual will become the target of an unexpected event, for example, a data breach, identity theft, or a cyber-attack (56). In the current study, perceived vulnerability refers to healthcare employees’ assessment of whether they are vulnerable to technology threats during digital transformation. If a user believes that the likelihood of being exposed to a cyber-attack is high due to the increasing reliance on technology in healthcare, they will be more careful to implement cybersecurity behavior, such as changing passwords regularly and activating two-factor authentication (58). According to some studies (47, 61), employees’ perceived vulnerability to cyber-attacks motivates them to adhere to cybersecurity regulations. This shows that perceived vulnerability has a significant impact on employees’ cybersecurity behavior and that those who perceive the level of vulnerability as high exhibit a higher level of cybersecurity behavior. Therefore, the current study hypothesizes that:

*H10*: Perceived vulnerability positively influences cybersecurity behavior during digital transformation.

### 3.8 Perceived severity

Perceived severity refers to an individual’s perception of the consequences of potential threats (47): the more severe an individual perceives a threat to be, the more likely he/she is to take preventive measures to mitigate the potential threat.

Employees’ perceptions of the severity of cyber risks significantly influence their safety concerns (60). Thus, perceived severity effectively reduces the misuse of information infrastructure. Research shows that perceived threat severity increases users’ motivation to engage in cybersecurity behavior to avoid these threats. In healthcare, when healthcare workers perceive that the threat of a data breach or health identity theft could lead to serious consequences, such as financial or psychological harm, they are more likely to adopt strong cybersecurity behavior (70). Kimpe et al. (47) demonstrated that concern about security threats led to a more positive attitude toward taking action, while Sulaiman et al. (60) showed that perceived threat severity has a positive effect on the implementation of security practices. Based on these findings, the following hypothesis is formulated:

*H11*: Perceived severity has a positive effect on cybersecurity behavior during digital transformation.

### 3.9 Self-efficacy

In PMT, self-efficacy refers to the extent to which individuals are confident in their ability to take preventive actions to protect themselves from security threats (56). In the healthcare context, if healthcare workers feel they have the skills to protect health records and systems, for example, by using data protection systems or implementing cyber best practice, they are more likely to adopt strong cybersecurity behavior. Several studies (15, 47, 60, 62) have confirmed a positive relationship between self-efficacy and individuals’ cybersecurity behavior. Therefore, the current study proposes the following hypothesis:

*H12*: Self-efficacy has a positive effect on cybersecurity behavior during digital transformation.

### 3.10 Response efficacy

Response efficacy refers to the extent to which individuals believe that the preventive measures they are taking will be effective in protecting themselves from threats (60). In the context of this study, response efficacy means the extent to which healthcare workers believe that the security measures they are taking will be successful in protecting their health and patient data from cyber threats, such as being hacked or leaked. Several studies (62) have shown a positive relationship between response efficacy and employee cybersecurity behavior. Therefore, the current study hypothesizes that:

*H13*: Response efficacy has a positive effect on cybersecurity behavior during digital transformation.

## 4 Methodology

### 4.1 Research instrument

The choice of methodology depends on the nature of the research problem, the researcher’s experience (71), and the research objectives (72). The current study used the exploratory quantitative approach for several reasons. With the study seeking to explore and understand the cybersecurity behavior of healthcare sector workers and to know the factors affecting their behavior, the application of quantitative methodology was consistent with these purposes. The quantitative approach was also suitable for testing hypotheses (71), one of the objectives of the study.

The study used the survey method to collect data, with this being a quantitative method for collecting accurate, valid, and reliable data in the research process (73).

In designing the scale items for the survey, the current study followed the guidelines found in the literature (74) to ensure the items’ validity and clarity. The questionnaire’s measurement items were developed from prior well-known studies to maintain construct and content validity, with some modifications made to achieve the study objectives (75). No strict rule governs the number of items that should constitute each construct, as mentioned by Hinkin (76). Each item in the questionnaire was assigned a unique code as shown in Appendix A, with the 11 dimensions comprising the following:

The privacy construct (*PRV*) was reflected by four items adopted from (56). Four items adopted from (56, 62) were used to measure perceived severity during digital transformation (*PS*). Trust in digital systems (*T*) was measured by four items adapted from (56). The three items to measure perceived vulnerability during digital transformation (*PV*) were adapted from (62). The current study also adapted four reflective items from (15, 47) to measure employee self-efficacy (*SE*). Response efficacy (*RE*) was measured by three items taken from (62). Certainty of punishment (*CP*) was measured by three items taken from (15). Measured complexity (*C*) by four items adapted from (58). Awareness (*AW*) was measured by four items adapted from (15, 19). Exploitability (*EX*) was measured by four items developed by the researcher. Finally, cybersecurity behavior during digital transformation (*CDT*) was measured by five reflective items adapted and modified from (19, 62). All measurement items that used 7-point Likert scales were assigned a serial number ranging from 1–7. The scales ranged from “1” for “strongly disagree” to “7” for “strongly agree” as suggested by (76).

A pre-test of the questionnaire was conducted to verify its validity (77). The draft questions and measures were sent to seven reviewers, two were professors specializing in cybersecurity and five were healthcare professionals. They examined the questionnaire format, items, structure, ease of use, and speed of completion. In addition, they provided some comments and recommendations for minor changes to improve the survey questionnaire.

### 4.2 Data collection

The questionnaire was designed using Google Forms. The research survey first clarified the objectives and purpose of the research on the introduction page, emphasizing the privacy of participants’ answers, as well as the approval of the Ethics Committee at Qassim. The first section comprised demographic questions which collected participants’ identification information. This was followed by several sections, each of which included items associated with each construct (78). The questionnaire was written in two languages, Arabic and English, to ensure that participants understood the questionnaire and to increase their response rate.

Choosing the appropriate sample was important in terms of achieving the study’s objectives through reliable and accurate results (73). The target population in this study comprised employees in the healthcare sector, for example, doctors, nurses, pharmacists, administrators, and others who used digital technologies, such as medical devices, health systems, and applications in their work. The study identified the target sample of healthcare workers for several reasons. Firstly, these employees were dealing directly with digital systems and sensitive data in their organizations. Therefore, they could measure the extent of the impact of these technologies on privacy and trust, as well as the severity and perception of threats associated with the use of digital systems. Secondly, Kamerer et al. (79) stated that nurses were considered the first line of defense against cyber-attacks, with most violations in the healthcare field related to the behavior and negligence of employees (80–82). Finally, these organizations are based on employees; therefore, measuring their cybersecurity behavior is extremely important. Accessing and analyzing information from healthcare workers would help to improve and enhance the secure digital transformation process and would build effective cybersecurity strategies.

The current study used the technique of snowball sampling to recruit participants. Snowball sampling is defined as sampling “through referrals between people who share or know others who have some characteristics of interest to the research” (83). It is a non-probability sampling technique that targets a specific population. It began with a small group that met the study criteria, who then referred to others with similar characteristics. The technique is suitable for this study due to the difficulty of obtaining a list of healthcare sector employees to target to measure their cybersecurity behavior during digital transformation (84).

The researcher calculated the appropriate sample size, that is, the number (*n*) of targeted participants, using the “10-times rule” (85). Most items in the current study led to the indicator cybersecurity behavior during digital transformation (*CDT*). Consequently, 50 participants (*n* = 50) were the minimum number needed for the sample (85). To gather the required number of responses for the current study, the researcher collected responses from 252 participants.

### 4.3 Method of data analysis

The study used dual analysis techniques: Partial Least Squares-based Structural Equation Modeling (PLS-SEM) and Artificial Neural Network (ANN) to accurately interpret the results. The current study employed the PLS-SEM technique due to its resilience to non-normal data distribution, ability to provide high statistical power (86), and effectiveness in analyzing complex structural models (87). The research model included more than 40 items and 10 constructs, making PLS-SEM an appropriate choice (88). This method has also been widely applied in recent cybersecurity healthcare studies (19, 58, 89). It enables researchers to explore theoretical extensions and evaluate models from a predictive perspective (56).

The current study used the PLS-SEM technique in a two-step method as proposed (85). The initial step was assessing the measurement model by evaluating the PLS-SEM results. The analysis was conducted to guarantee the validity and reliability of the construct measurements. To determine the relevance of path coefficients (hypotheses testing), the second step assessed the structural models that explained the relationships between the latent variables (independent and dependent variables).

The study used an artificial neural network (ANN) as a supplementary method to re-examine and analyze the research model. This method is characterized by its ability to analyze complex relationships (90), both linear and nonlinear, and provides accurate predictive results compared to traditional methods such as linear regression (91). It is also capable of handling issues caused by inadequate information. Moreover, many studies (92–94) have used an ANN to examine the relationships between variables in the context of cybersecurity and digital transformation research.

The research relied on the results of PLS-SEM analysis to identify important variables, which were used as inputs in the ANN analysis to enhance the study results. The ANN analysis was performed using IBM SPSS Statistics version 30.

### 4.4 Pilot study

A pilot study is an exploratory study conducted on a small sample of the target research population before implementing the main study, with the aim of testing the tools and procedures, while effectively and efficiently ensuring the applicability of the main study (95). In the current study, a pilot study was conducted to test the reliability and validity of the measurement instrument (i.e., the questionnaire) and to confirm the applicability of the proposed hypotheses and analytical procedures on a small scale before the main data collection. The pilot study sample comprised 124 participants from the targeted sample. The data were analyzed using PLS-SEM to assess reliability and structural validity. The initial results showed that some items had weak indicators; thus, the research instrument was modified by deleting items with weak loadings.

## 5 Results

### 5.1 Sample’s characteristics

The descriptive statistics of the study’s sample provided a clear view of the distribution of participants based on demographic variables. The demographic characteristics of participants, all of whom worked in various health sector jobs, were collected through the survey, including gender, age group, job title, and years of experience, as shown in Table 2. According to the results, 55.6% (*n* = 112) of the participants were female, while 44.4% (*n* = 140) were male. The result showed a higher frequency of male participants compared to female participants in this study. Moreover, analysis showed that the largest group of participants was within the age group of 30–40 years (46.03%; *n* = 116), followed by the age group of 40–50 years (28.57%; *n* = 72), while 19.05% (*n* = 48) were in the age group of 18–30 years, indicating a good representation of youth in the sample. The least represented group comprised those aged over 50 years at 6.35% (*n* = 16).

**Table 2:** Demographic statistics.

Demographics	Category	Frequency	Percentage
Gender	Female	112	44.4
	Male	140	55.6
Age	18–30 years	48	19.05
	30–40 years	116	46.03
	40–50 years	72	28.57
	More than 50 years	16	6.35
Job role	Doctor	54	21.43
	Nurse	55	21.83
	Pharmacist	30	11.90
	Other	113	44.84
Number of years of work experience	0–2 years	32	12.70
	2–4 years	25	9.92
	4–6 years	45	17.86
	More than 7 years	150	59.52

As for job roles, the study included all job roles in healthcare. Data showed that nurses represented the largest group of participants with 21.83% (*n* = 55), followed by doctors (21.43%; *n* = 54), while pharmacists comprised 11.90% (*n* = 30). The option of “other” was available due to the great diversity of professional roles in the health sector. This category comprised 44.84% (*n* = 113) of participants and included specialists, such as epidemiologists, therapeutic nutritionists, and social workers, as well as technicians, such as laboratory technicians, radiology technicians, etc. Most participants (59.52%; *n* = 150) had more than 7 years of experience, followed by participants with 4–6 years of experience (17.86%; *n* = 45), then participants with 0–2 years of experience (12.70%; *n* = 32), and, finally, participants with 2–4 years of experience (9.92%; *n* = 25).

### 5.2 Measurement model evaluation

**Figure 2:**
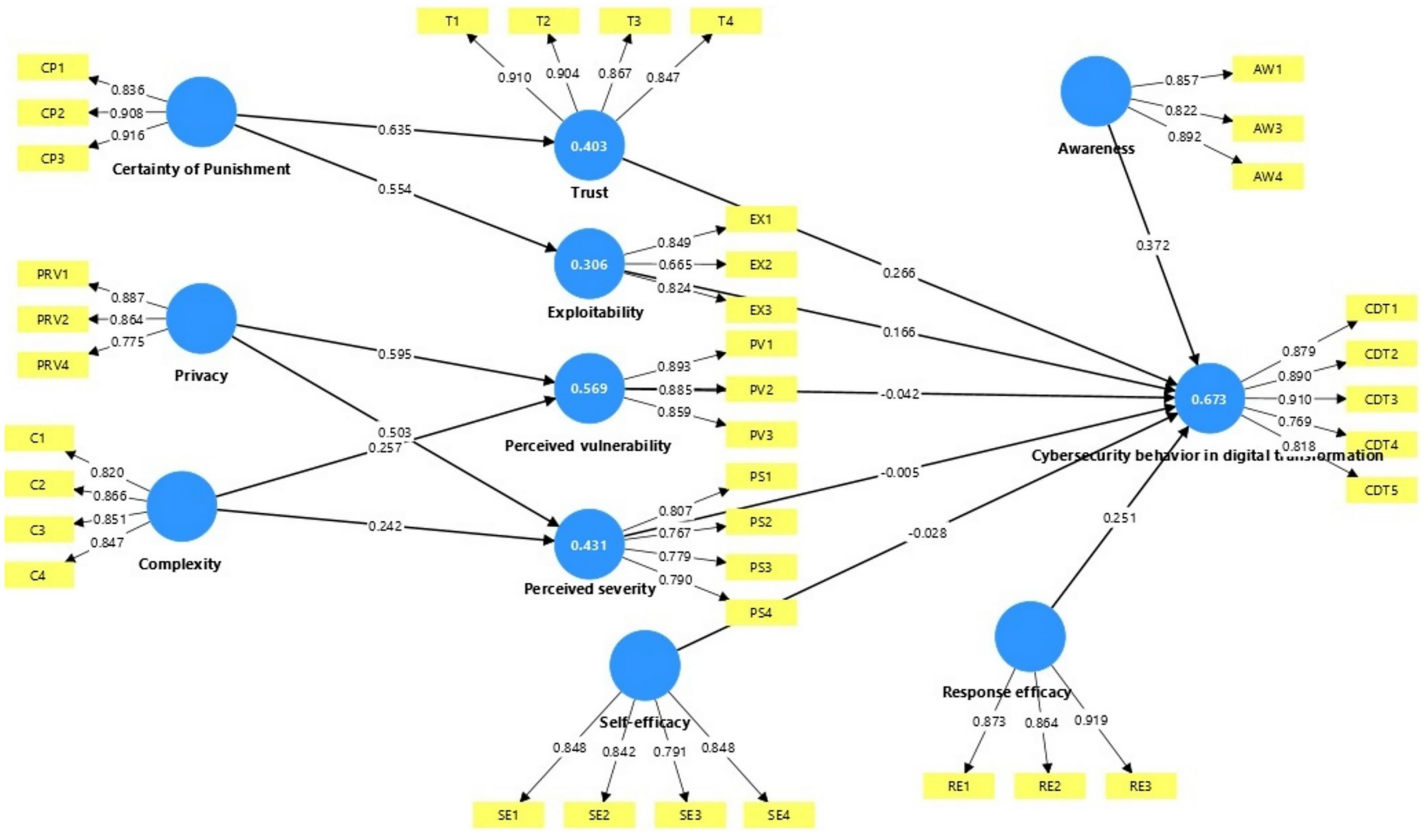
Measurement model results.

Figure 2 presents the results of the measurement model through the first step of the analysis by the PLS-SEM algorithm. The measurement models were evaluated, following Hair et al. (85), with four indicators: reliability, internal consistency reliability, convergent validity, and discriminant validity to ensure the validity and reliability of the measurements of the latent variables. The results of each of these indicators are discussed separately in the following subsections.

#### 5.2.1 Assessment of indicator reliability

Measuring the reliability of indicators is one of the basic criteria in evaluating the measurement model, as reliability reflects the extent to which the element can accurately and consistently measure the latent variable. For indicators to be evaluated as being reliable, the weights of indicators must be greater than 0.7, according to (85). The weights of indicators for the current study ranged from 0.7–0.9 as shown below in Table 3, indicating a high level of reliability for all indicators, except for indicator *EX2*, which, at 0.665, it is still significantly higher than the minimum value of 0.50, suggested by Fornell and Larcker (96).

**Table 3:** Reliability and validity of construct.

Construct	Items	Loading	Cronbach’s alpha	Composite reliability (rho_A)	Composite reliability (rho_c)	Average variance extracted (AVE)
Awareness	*AW1*	0.857	0.820	0.828	0.893	0.735
	*AW3*	0.822				
	*AW4*	0.892				
Complexity	*C1*	0.820	0.868	0.874	0.910	0.716
	*C2*	0.866				
	*C3*	0.851				
	*C4*	0.847				
Cybersecurity behavior during digital transformation	*CDT1*	0.879	0.907	0.911	0.931	0.731
	*CDT2*	0.890				
	*CDT3*	0.910				
	*CDT4*	0.769				
	*CDT5*	0.818				
Certainty of punishment	*CP1*	0.836	0.865	0.878	0.917	0.787
	*CP2*	0.908				
	*CP3*	0.916				
Exploitability	*EX1*	0.849	0.707	0.782	0.825	0.614
	*EX2*	0.665				
	*EX3*	0.824				
Privacy	*PRV1*	0.887	0.795	0.795	0.881	0.712
	*PRV2*	0.864				
	*PRV4*	0.775				
Perceived severity	*PS1*	0.807	0.796	0.802	0.866	0.618
	*PS2*	0.767				
	*PS3*	0.779				
	*PS4*	0.790				
Perceived vulnerability	*PV1*	0.893	0.853	0.857	0.911	0.773
	*PV2*	0.885				
	*PV3*	0.859				
Response efficacy	*RE1*	0.873	0.862	0.864	0.916	0.785
	*RE2*	0.864				
	*RE3*	0.919				
Self-efficacy	*SE1*	0.848	0.853	0.859	0.901	0.694
	*SE2*	0.842				
	*SE3*	0.791				
	*SE4*	0.848				
Trust	*T1*	0.910	0.905	0.905	0.933	0.778
	*T2*	0.904				
	*T3*	0.867				
	*T4*	0.847				

#### 5.2.2 Assessment of internal consistency reliability

Several metrics are offered by PLS-SEM to confirm the validity of a construct’s internal consistency. Firstly, according to studies by (97, 98), Cronbach’s alpha (*α*) coefficient should have a minimum acceptable value of 0.60 or 0.70 and a maximum acceptable value of 0.95. All Cronbach’s alpha values in the current study’s results, as shown in Table 3, were between these acceptable levels, indicating strong reliability.

Secondly, internal consistency reliability is measured by looking at the composite reliability rho_c values. Typically, rho_c values between 0.60 and 0.70 indicate an acceptable level of reliability, while results ranging from 0.7–0.95 indicate excellent to good reliability levels (85). Values above 0.95 are considered problematic (99). In the current study, Table 3 shows that the composite reliability rho_c results for each construct were between 0.933 and 0.825, thus exceeding the cut-off value of 0.70.

Finally, a rho_A value of 0.70 or more is considered to signify composite reliability (87). As it usually lies in the middle of the values for Cronbach’s alpha and composite reliability rho_c, the rho_A reliability measure is thought to be a good compromise between the two (85).

#### 5.2.3 Assessment of convergent validity

Convergent validity is measured using the average variance extracted (AVE) value, as suggested by (87). Hair et al. (85) stated that the average variance extracted (AVE) value should be 0.5 or higher to ensure that the construct explains 50% or more of the total variance of its indicators. As presented in Table 3, the results of the convergent validity assessment indicate that all the AVE values exceed the recommended threshold. Thus, these results demonstrated adequate convergent validity.

#### 5.2.4 Assessment of discriminant validity

Two metrics are available for evaluating discriminant validity. As suggested by Fornell and Larcker (96), the first metric is the Fornell–Larcker criterion, which evaluates discriminant validity by contrasting the relationships between different factors. As recommended in (97), the square root of each AVE should have a value greater than the highest correlation between that construct and any other construct. The AVE square root values in Table 4 are represented by the diagonal numbers in bold font, while the other values represent correlations. Table 4 indicates that each construct has sufficient discriminant validity, as the square root value of its AVE is higher than the correlations between the variables.

**Table 4:** Fornell–Larcker criterion.

Constructs	*AW*	*C*	*CDT*	*CP*	*EX*	*PRV*	*PS*	*PV*	*RE*	*SE*	*T*
*AW*	**0.857**										
*C*	0.278	**0.846**									
*CDT*	0.677	0.172	**0.855**								
*CP*	0.483	0.177	0.602	**0.887**							
*EX*	0.445	0.438	0.523	0.554	**0.784**						
*PRV*	0.273	0.489	0.117	0.146	0.357	**0.844**					
*PS*	0.341	0.488	0.245	0.235	0.390	0.621	**0.786**				
*PV*	0.238	0.548	0.129	0.216	0.357	0.721	0.665	**0.879**			
*RE*	0.461	0.087	0.674	0.634	0.433	0.058	0.213	0.093	**0.886**		
*SE*	0.545	0.139	0.557	0.548	0.355	0.094	0.229	0.154	0.653	**0.833**	
*T*	0.538	0.116	0.706	0.635	0.412	0.008	0.151	0.030	0.764	0.631	**0.882**

The diagonal values in bold represent the square root of the AVE for each construct.

Prior research (85, 100) has indicated that, in some cases, Fornell–Larcker’s measure may be inadequate. Therefore, Henseler et al. (97) proposed another measure of discriminant validity, namely, the heterotrait–monotrait (HTMT) ratio, which should not exceed a threshold value above 0.90 to obtain adequate discriminant validity. As shown in Table 5, all HTMT ratio values are less than 0.90; thus, discriminant validity is determined.

**Table 5:** HTMT ratio.

Constructs	*AW*	*C*	*CDT*	*CP*	*EX*	*PRV*	*PS*	*PV*	*RE*	*SE*	*T*
*AW*											
*C*	0.337										
*CDT*	0.780	0.200									
*CP*	0.571	0.219	0.676								
*EX*	0.576	0.648	0.604	0.648							
*PRV*	0.340	0.578	0.146	0.183	0.538						
*PS*	0.431	0.560	0.301	0.301	0.551	0.763					
*PV*	0.285	0.630	0.148	0.261	0.522	0.871	0.792				
*RE*	0.547	0.129	0.759	0.727	0.485	0.081	0.280	0.119			
*SE*	0.646	0.164	0.630	0.632	0.417	0.134	0.291	0.183	0.756		
*T*	0.620	0.137	0.777	0.710	0.453	0.048	0.194	0.051	0.863	0.713	

### 5.3 Structural model evaluation

**Figure 3:**
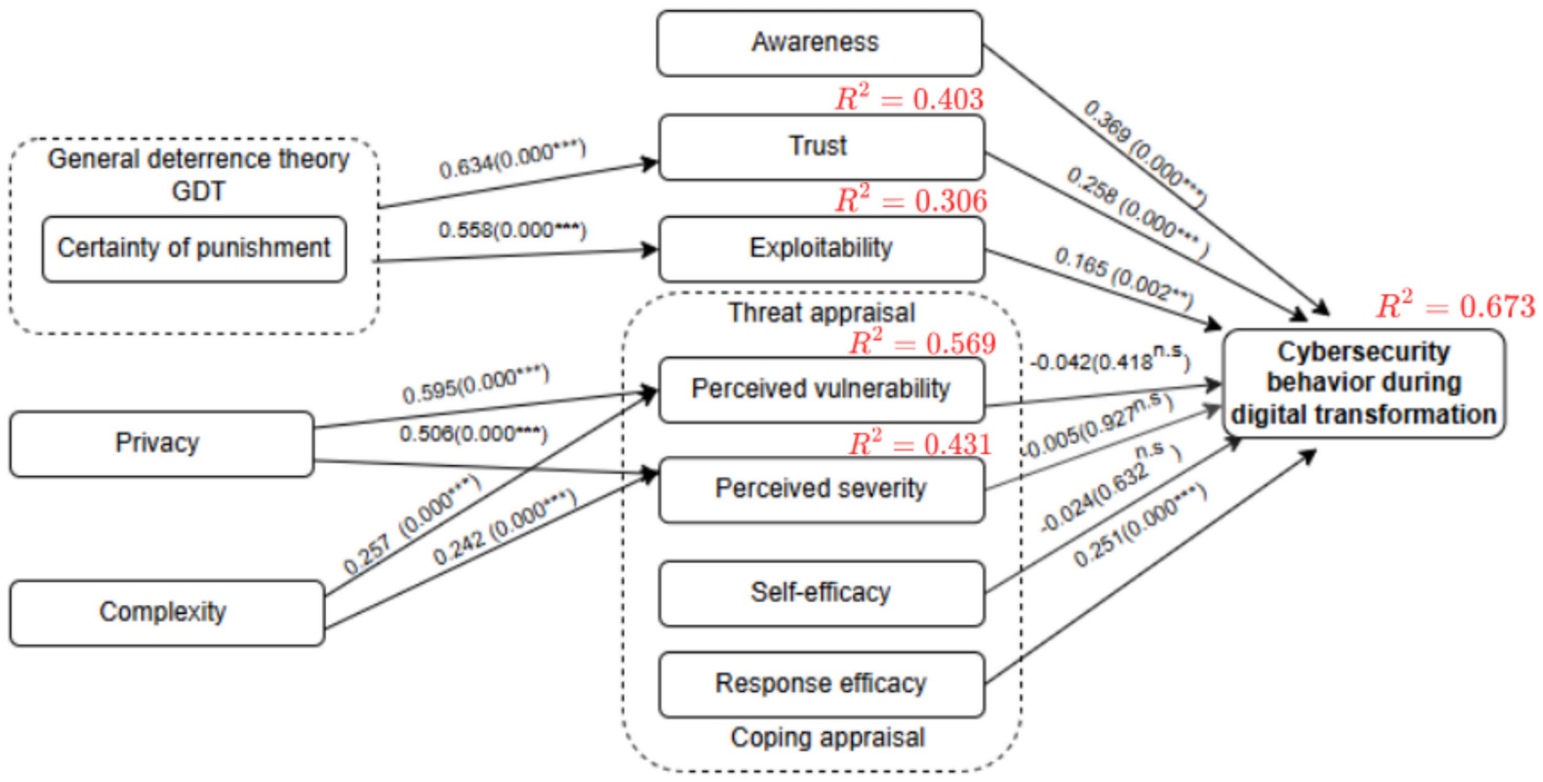
Structural model results. n.s. = non-significant; ^∗^*p* < 0.05; ^∗∗^*p* < 0.01; ^∗∗∗^*p* < 0.001.

The evaluation of the structural model outputs is the second stage of the PLS-SEM investigation. In accordance with Hair et al. (85), the structural model in the current study was evaluated using the following standard measures: collinearity was evaluated first, followed by path coefficients, coefficients of determination (*R^2^* value) effect size (*f^2^* value), and predictive significance (*Q^2^*). Figure 3 presents the results of the structural model evaluation. These results are discussed in the following subsections, along with an analysis of the extent to which the results agree with the research hypotheses and their impact on the interpretation of the relationships between the variables.

#### 5.3.1 Assessment size and significance of path coefficients

In the current study, path coefficients were determined in the structural model evaluation stage, in which the significance of the proposed hypotheses were identified and examined and the relationships between external and internal constructs were analyzed. The study used bootstrapping analysis to determine the path coefficients and the constructs’ level of statistical significance. Hair et al. (85) defined bootstrapping as a “resampling approach that draws random samples (with replacement) from the data and uses these samples to estimate the path model several times under slightly changed data constellations.” In addition, the bootstrapping process generated the *p*-values and *t*-statistic values to investigate the statistical significance and relevance (i.e., the size) of the path coefficient. Nunnally and Bernstein (101) contended that these values should be as follows: for a 5% significance level, *t*-values must be more than 1.96 (two-tailed), 2.68 for a 1% significance level, and 3.29 for a 0.1% significance level. To assure the stability of the results, the current study used 10,000 bootstrap samples, the quantity suggested in (102–104).

Table 6 provides a summary of the results of the path analysis and hypotheses testing, as previously shown in Figure 2. Of the 13 relationships in the study’s research model directly examined by hypotheses, the results showed that three were not statistically significant. Moreover, the results showed a positive relationship between certainty of punishment (*CP*) and trust (*T*) (*β* = 0.635; *t* = 12.126; *p* = 0.000), which supported H1. Certainty of punishment (*CP*) was also found to have a positive relationship with exploitability (*EX*) (*β* = 0.554; *t* = 11.368; *p* = 0.000); therefore, H2 was supported. The study results indicated that privacy (*PRV*) was positively associated with perceived vulnerability (*PV*) (𝛽=0.595; *t* = 11.701; *p* = 0.000), supporting H3, and had a positive effect on perceived severity (*PS*) (𝛽=0.503; *t* = 9.704; *p* = 0.000), supporting H4.

**Table 6:** Results of hypotheses testing.

Hypotheses	Association	Original sample (O)	Sample mean (M)	Standard deviation (St. Dev.)	t-statistics (|O/St. Dev.|)	*p*-values	Supported
H1	CP → T	0.635	0.634	0.052	12.126	0.000***	Yes
H2	CP → EX	0.554	0.558	0.049	11.368	0.000***	Yes
H3	PRV → PV	0.595	0.595	0.051	11.701	0.000***	Yes
H4	PRV → PS	0.503	0.506	0.052	9.704	0.000***	Yes
H5	C → PV	0.257	0.257	0.055	4.658	0.000***	Yes
H6	C → PS	0.242	0.242	0.060	4.014	0.000***	Yes
H7	AW → CDT	0.372	0.369	0.054	6.890	0.000***	Yes
H8	T → CDT	0.266	0.258	0.075	3.525	0.000***	Yes
H9	EX → CDT	0.166	0.165	0.052	3.174	0.002**	Yes
H10	PV → CDT	−0.042	−0.044	0.052	0.809	0.418	No
H11	PS → CDT	−0.005	−0.001	0.057	0.091	0.927	No
H12	SE → CDT	−0.028	−0.024	0.059	0.479	0.632	No
H13	RE → CDT	0.251	0.256	0.071	3.550	0.000***	Yes

** *p* < 0.01; *** *p* < 0.001.

The study also found that complexity (*C*) had a positive effect on perceived vulnerability (*PV*) (𝛽=0.257; *t* = 4.658; *p* = 0.000) and also had a positive effect on perceived severity (*PS*) (𝛽=0.242; *t* = 4.014; *p* = 0.000); thus, these results supported H5 and H6. The effect of awareness (*AW*) on employees’ cybersecurity behavior during digital transformation (*CDT*) was positive and statistically significant (𝛽=0.372; *t* = 6.890; *p* = 0.000), supporting H7. In addition, trust (*T*) had a positive effect on cybersecurity behavior during digital transformation (*CDT*) (𝛽=0.266; *t* = 3.525; *p* = 0.000), confirming H8. The results also showed that exploitability (*EX*) positively affected cybersecurity behavior during digital transformation (*CDT*) (𝛽=0.166; *t* = 3.174; *p* = 0.002), with this finding supporting H9.

However, no statistically significant effect was found for perceived vulnerability (*PV*) on cybersecurity behavior during digital transformation (*CDT*) (𝛽= − 0.042; *t* = 0.809; *p* = 0.418), and no significant effect was found for perceived severity (*PS*) on cybersecurity behavior during digital transformation (*CDT*) (𝛽= − 0.005; *t* = 0.091; *p* = 0.927), which led to the rejection of H10 and H11. It was found that self-efficacy (*SE*) did not have a statistically significant effect on employees’ cybersecurity behavior during digital transformation (*CDT*) (𝛽= − 0.028; *t* = 0.479; *p* = 0.632), which led to the rejection of H12. Finally, the results indicated that response efficacy (*RE*) had a positive impact on cybersecurity behavior during digital transformation (*CDT*) (𝛽=0.251; *t* = 3.550; *p* = 0.000), confirming H13.

#### 5.3.2 Assessment of coefficients of determination (*R*^2^ values)

Shmueli and Koppius (105) stated that the *R^2^* value, also known as the coefficient of determination (104), quantifies the variance explained in each of the endogenous constructs and, thus, the explanatory power of the model. Additionally, the model’s explanatory power rises with increasing *R^2^* values, with 0.75, 0.50, and 0.25 being regarded as substantial, moderate, and weak, respectively (85).

As shown in Table 7, the model showed substantial predictive accuracy for cybersecurity behavior during digital transformation (*R*^2^ = 0.673). Perceived vulnerability (*R*^2^ = 0.569), perceived severity (*R*^2^ = 0.431), and trust (*R*^2^ = 0.403) demonstrated moderate predictive accuracy, while exploitability had weak predictive accuracy (*R*^2^ = 0.306). These results indicate varying levels of explanatory power across the model’s constructs.

**Table 7:** *R*^2^, *Q*^2^, predictive relevance, and effect size ( $f^{2}$ ).

Endogenous variables	$R^{2}$	$Q^{2}$	Exogenous variables	$f^{2}$	Effect size
*CDT*	0.673	0.608	*AW*	0.240	Medium
			*EX*	0.055	Small
			*PS*	0.000	No effect
			*PV*	0.003	No effect
			*RE*	0.068	Small
			*SE*	0.001	No effect
			*T*	0.075	Small
*EX*	0.306	0.295	*CP*	0.442	Large
*PS*	0.431	0.418	*PRV*	0.339	Large
			*C*	0.078	Small
*PV*	0.569	0.558	*PRV*	0.626	Large
			*C*	0.117	Medium
*T*	0.403	0.395	*CP*	0.674	Large

#### 5.3.3 Assessment of effect size (f^2^) value

The effect size measure is used to assess the effect of removing a particular exogenous construct from the model based on the *R^2^* value of the endogenous construct. As recommended by one study (96), effect sizes are found to be low, medium, and high at *f^2^* values of 0.02, 0.15, and 0.35, respectively: a value below 0.02 shows no effect.

As shown in Table 7, *PS, PV,* and *SE* had no effect size (*f^2^*) value on *CDT*, whereas awareness (*AW*) had a medium effect with an *f^2^* value of 0.240 on *CDT*, while exploitability (*EX*) (*f^2^* = 0.055), response efficacy (*RE*) *f^2^* = 0.068, and trust (*T*) *f^2^* = 0.075 showed a small effect on the *CDT* variable. In addition, the results indicated that privacy (*PRV*) had a significant effect on both perceived severity (*PS*) (*f^2^* = 0.339) and perceived vulnerability (*PV*) (*f^2^* = 0.626), while complexity (*C*) showed a small effect on *PS* (*f^2^* = 0.078) and a medium effect on *PV* (*f^2^* = 0.117). Certainty of punishment (*CP*) showed a significant effect on exploitability (*EX*) (*f^2^*) = 0.442 and it also had a very significant effect on trust (*T*) (*f^2^* = 0.674).

#### 5.3.4 Assessment of predictive relevance (*Q*^2^ value)

The *Q^2^* value evaluates the predictive relevance of the endogenous constructs or the predictive capability of the PLS path model (85). The PLSpredict algorithm was used to calculate the *Q^2^* metric in SmartPLS. The number of folds (*K* = 10) and repetitions (*r* = 10) in the current study’s training sample were in accordance with the number recommended in the study by (106), and exceeded the minimal sample criteria. A *Q^2^* value of 0 (zero) or less denotes the lack of predictive relevance for endogenous constructs, whereas a *Q^2^* value greater than 0 (zero) suggests that the model has predictive relevance (88, 96). Table 7 shows that all the endogenous constructs had *Q^2^* values greater than 0 (zero); thus, a highly predictive relevance model was created by the current study. The *Q^2^* value of *CDT* was 0.608, with this value indicating that the exogenous constructs (*T, EX, PV, PS, SE*, and *RE*) had strong predictive relevance over the endogenous construct (*CDT*).

### 5.4 Artificial neural network (ANN)

**Figure 4:**
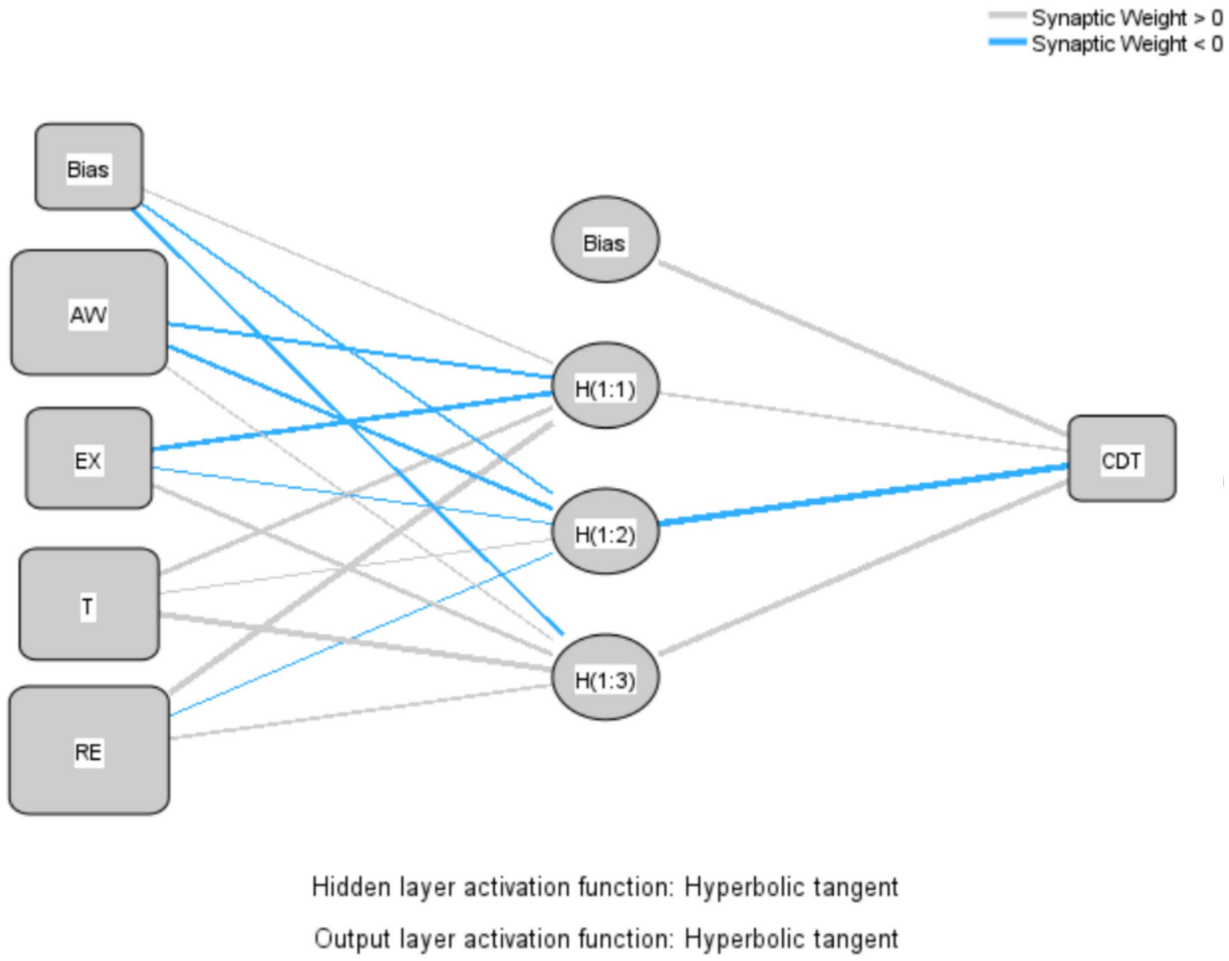
Artificial neural network (ANN) model.

To ascertain the relative significance of the relationships of exogenous variables to an endogenous variable and prove the results of the PLS analysis, the current study employed a multi-layer perceptron artificial neural network (ANN) using a feed-forward back-propagation (FFBP) method. The ANN algorithm can learn to predict the results of an analysis by using a FFBP method in which inputs are sent forward and estimated errors are sent backward (107). The current study utilized IBM SPSS Statistics (SPSS) v.30 software to conduct the ANN analysis, following procedures in previous studies (108, 109). In the ANN model, important hypothesized predictors are used as ANN inputs (110); hence, *AW, EX, T,* and *RE* were selected as the independent variables whose importance and influence on *CDT* were proven by PLS results. These independent variables formed part of the input layer (neurons), while *CDT* was part of the output layer, as shown in Figure 4. Tenfold cross-validation was applied to the data set to avoid overfitting, producing 10 ANN models. In terms of the data, 70% was used for training, while 30% was utilized for testing to determine the predicted accuracy of the trained network. Furthermore, the algorithm produced a specified number of hidden neurons, with the hyperbolic tangent activation function used to activate both the hidden layer and output layer. To assess the predictive accuracy of the study’s research model, the RMSE was computed for each network in the ANN model in compliance with multiple studies (92, 94, 107). As shown in Table 8, the ANN model’s mean RMSE for training data was 0.202, while it was 0.201 for testing data. The lower RMSE number denoted a more accurate fit and forecast of the data, as well as suggesting a degree of predictive accuracy. In addition, the number of hidden neurons in an ANN model with non-zero synaptic weights were used to evaluate the significance of external variables.

**Table 8:** Root mean square error (RMSE) values for training and testing.

Network	Sum of square error (Training)	Sum of square error (Testing)	RMSE (Training)	RMSE (Testing)
1	6.419	3.591	0.191	0.217
2	8.024	2.006	0.207	0.176
3	7.041	3.326	0.203	0.203
4	6.505	3.5	0.196	0.207
5	6.763	3.004	0.197	0.196
6	9.036	2.05	0.222	0.172
7	7.415	2.88	0.211	0.184
8	6.616	3.629	0.196	0.214
9	6.036	5.08	0.182	0.269
10	8.349	2.087	0.215	0.170
Mean	7.220	3.115	0.202	0.201
Std. Dev.	0.967	0.945	0.0121	0.0296

Sensitivity analysis was performed to statistically evaluate the predictive capabilities of the exogenous variables in relation to the endogenous variable after determining the expected accuracy and predictive importance of the ANN model (111, 112). The relative importance of each exogenous variable was determined, and the standardized relative value was calculated, as shown in Table 9. When analyzing the four variables in the ANN model, awareness (*AW*) was the most important and strongest predictor of employees’ cybersecurity behavior during digital transformation (*CDT*), with a standardized relative importance of 94.1%, followed by trust (*T*) with a standardized importance of 81.9%. However, the relative importance of *CDT* was less well predicted by response efficacy (*RE*) (66.45%) and exploitability (*EX*) (35.06%), in that order. As expected, awareness (*AW*) was the most reliable predictor of *CDT*, while *EX* was the least important. The study’s results found agreement between the results of the ANN and PLS-SEM analyses, in accordance with the importance of each variable in the study model, as shown in Table 10.

**Table 9:** Sensitivity analysis.

Network	*AW*	*EX*	*T*	*RE*
1	0.284	0.223	0.18	0.312
2	0.35	0.183	0.278	0.19
3	0.279	0.197	0.234	0.29
4	0.345	0.167	0.222	0.267
5	0.309	0.148	0.329	0.214
6	0.326	0.195	0.219	0.26
7	0.347	0.137	0.331	0.185
8	0.321	0.188	0.33	0.161
9	0.257	0.124	0.407	0.212
10	0.357	0.16	0.264	0.219
Average relative importance (RI)	0.3175	0.1722	0.2794	0.231
Normalized RI (%)	94.1%	51.7%	81.9%	69.2%

**Table 10:** Comparison of ANN results and PLS-SEM results.

Independent variables	PLS-SEM (path coefficient)	Ranked	ANN (Normalized importance) (%)	Ranked
AW	0.372	1	94.1%	1
T	0.266	2	81.9%	2
RE	0.251	3	69.2%	3
EX	0.166	4	51.7%	4

Consequently, the current study offers helpful insights about the relative significance of awareness, trust, response efficacy, and exploitability as important indicators of employee cybersecurity behavior during digital transformation. Therefore, the current study is consistent with previous studies (91, 92) in that the results of the hybrid analysis are identical, indicating greater confidence in the validity of the research model.

### 5.5 Common method bias (CMB)

Statistical techniques were used in the current study to evaluate the potential for common method bias (CMB). Firstly, a procedural remedy was created, in line with that used in the study by (113). This technique, applied during the pre-test phase to make the scale items clearer and to prevent any ambiguity, occasionally interspersed the pattern of questions rated on Likert scales with multiple-choice questions. Secondly, Harman’s single-factor test (SFT) was used through exploratory factor analysis (EFA) in the SPSS software. The results showed that the first factor explained 27.77% of the total variance, which is below the 50% threshold. Therefore, CMB was not a major concern in this study (113).

## 6 Discussion

### 6.1 Certainty of punishment

The general deterrence theory (GDT) is considered one of the valuable theories that can be applied in relation to cybersecurity during digital transformation to prevent risky behavior by imposing clear and strict penalties and enhancing cybersecurity behavior among employees (114). Consequently, the study’s results supported all hypotheses related to the factor of certainty of punishment. Interestingly, the results found that certainty of punishment had a significant impact on trust, as the path coefficient value and *t*-value reached 0.000 and 12.126, respectively. In addition, certainty of punishment had an impact on exploitability, as the path coefficient value and *t*-value reached 0.000 and 11.368; thus, these results are consistent with those of previous studies (51, 54). This explains that certainty of punishment plays a crucial role in shaping employees’ cybersecurity behavior in the healthcare sector, as it enhances employees’ trust in cybersecurity. It also reduces the digital systems’ vulnerability to exploitation, thus contributing to improving cybersecurity protection during digital transformation in the healthcare environment.

### 6.2 Privacy

Privacy is gaining increasing importance in the digital age, especially in sensitive sectors, such as healthcare, where electronic records contain highly sensitive data (115). With the rise of cyber threats, such as cyber-attacks and commercial exploitation of data, individuals are becoming more aware of the risks of privacy violation, increasing their perception of the severity of the threat and their potential vulnerability (116).

This study’s results showed that privacy positively affects the severity of perceived threats (*t* = 9.704, *p* = 0.000), and that it also positively affects perceived vulnerability (*t* = 11.701, *p* = 0.000), indicating that individuals who care about their privacy view privacy violations as a serious threat and feel more vulnerable to the associated threats. This finding is supported by a prior study (56) which indicated that individuals who are more aware of privacy are more aware of the consequences of its violation.

### 6.3 Complexity

The results of the current study found a statistically significant positive effect between complexity and perceived vulnerability in the healthcare environment, with a *t*-value of 4.658 and a *p*-value of 0.000. This indicates that increasing the complexity of digital systems leads to an increase in employees’ perception of the extent to which systems are vulnerable to cyber threats. A statistically significant positive relationship was also found between complexity and the perceived severity of threats, as evidenced by the path coefficient value and *t*-value of 0.000 and 4.014, respectively. This means that increasing the level of system complexity leads to an increase in employees’ perception of the severity of security risks that may result from attacks.

Despite the increasing importance of cybersecurity in the healthcare environment, no previous studies have directly examined the relationship between complexity and threat appraisal, either in terms of perceived vulnerability or perceived severity of threats.

Previous studies (58, 59) have shown that increasing the complexity of digital systems hinders their successful adoption, impacting their efficiency and security. In this context, the results of the current study confirm an additional dimension to this effect and provide new empirical evidence. The study indicates that complexity not only hinders the adoption of digital systems in the healthcare environment, but also increases employees’ perceptions of security risks and perceived vulnerability which may lead to increased concerns about protecting data and systems from cyber threats.

### 6.4 Awareness

As posited in the study’s H7, the results showed that awareness had a significant relationship with the cybersecurity behavior of employees in the healthcare sector, with a path coefficient of 0.000 and a *t*-value of 6.890. This result was consistent with the findings of (19, 116), showing that awareness of risk is the most influential factor on cybersecurity, as it drives individuals to adopt strong security practices to protect digital systems. Similarly, the prior study in (82) showed the importance of awareness, with more than 50% of healthcare employees aware of the existence of antivirus software and the importance of locking their devices when leaving them, while 76% confirmed that following cybersecurity policies helped them to better perform their jobs. In addition, the current study is in agreement with a previous study (117) that examined the impact of awareness of cyber-attacks and hacking on customers’ awareness of cybersecurity in relation to digital transformation in the banking sector.

Therefore, awareness is the first influential factor in shaping employees’ cybersecurity behavior, according to the results of these analyses. This indicates that raising awareness can lead to improved compliance with security practices, which reduces cyber risks and enhances the overall protection of systems and data.

### 6.5 Trust

The results of the study’s statistical tests confirmed support for H8, showing the positive effect of trust on employees’ cybersecurity behavior during digital transformation, with a path coefficient value of 0.266 and a *t*-value of 3.525 at a significance level of 0.000. These results are in line with previous studies (64, 66) which explained that, when employees or patients feel confident that modern digital technologies, such as the Internet of Things (IoT) (63), are well protected, they have fewer concerns about privacy violations or exposure to cyber-attacks (118). This enhances their commitment to the cybersecurity policies of these systems. As employees trust the systems and technologies, they rely on them and are more willing to comply with the required security practices (65). These results reflect the importance of building and enhancing trust in digital systems to ensure employees’ commitment to security practices.

### 6.6 Exploitability

The results of the study supported H9, confirming the existence of a positive relationship between exploitability and employees’ cybersecurity behavior during digital transformation, with an effect value of 0.166 at a significance level of 0.002. This positive effect can be explained by the point that when employees perceive that the digital systems with which they work are vulnerable to exploitation, this may lead to a significant increase in their cybersecurity behavior and to taking precautionary measures to reduce risks, such as using strong passwords, activating antivirus programs, and performing continuous updates.

Most studies (12, 22, 68) have focused on exploitability from a technical perspective without addressing its impact on employees’ cybersecurity behavior. Hence, the current study provides a new theoretical contribution in this regard. It is the first study of its kind to empirically examine the relationship between exploitability and employees’ cybersecurity behavior in the healthcare sector.

### 6.7 Perceived vulnerability

The study’s H10 stated that “perceived vulnerability positively influences cybersecurity behavior during digital transformation.” The study’s results did not support this hypothesis, as the coefficient value was −0.042 with a *p*-value of 0.418, which is not statistically significant. This indicates that perceived vulnerability has no significant effect on employees’ cybersecurity behavior during digital transformation. These results are in line with previous studies (56, 60, 62), with their findings that perceived vulnerability does not have a significant effect on cybersecurity behavior.

These results explain employees’ trust in digital technologies, as employees may feel that the security systems and procedures implemented in their organization are strong enough to protect them, which reduces the impact of their perception of vulnerability on their cybersecurity behavior.

In contrast, previous research (47, 119) has shown a significant relationship between perceived vulnerability and the intention to adopt online security measures. One explanation is that one of these previous studies (47) did not focus on a specific sector, in which cyber risks and crimes were more prominent thus increasing individuals’ awareness and willingness to take cybersecurity measures. Conversely, the current study focused on healthcare employees during digital transformation. These employees felt that their organizations provided strong protection, which reduces the impact of perceived vulnerability on their cybersecurity behavior and, thus, the relationship between perceived vulnerability and employees’ cybersecurity behavior was found to be insignificant.

### 6.8 Perceived severity

The current study’s results also found that H11 was not supported, as the relationship coefficient was −0.005 with a *p*-value of 0.927, indicating no significant positive effect of threat severity on employees’ cybersecurity behavior during digital transformation in the healthcare sector. These results are consistent with similar findings in (56, 62) which indicated that perceived threat severity on its own was not sufficient to promote cybersecurity behavior. The explanation was that the effect of threat severity is weakened when employees feel that the organization provides a strong protective environment, which prompts them to rely on institutional systems instead of taking additional personal protective measures. In contrast, previous studies (47, 60, 119) found a significant effect of perceived threat severity, as these researchers found that threat severity prompts individuals to adopt additional forms of cybersecurity behavior.

### 6.9 Self-efficacy

The study’s results did not support the relationship between self-efficacy and cybersecurity behavior during digital transformation. The value of the effect coefficient was −0.028 with a *p*-value of 0.632, indicating no statistically significant effect. These results are consistent with Lee et al. (46) who showed that the effect of self-efficacy may not be direct or strong when measured alone, especially in work environments such as the healthcare sector, where institutional support, security training, and organizational culture play a greater role in motivating cybersecurity behavior. However, some previous studies (47, 62, 119) found results that conflicted with those of the current study. These studies found that self-efficacy had an effect on employee cybersecurity behavior, with individuals who were confident in their ability to deal with cybersecurity threats more willing to take preventive measures.

### 6.10 Response efficacy

The study results showed strong support for H13, with a path coefficient of 0.251 and a *p*-value of 0.000, indicating a statistically significant positive effect. The study results are consistent with those of previous studies (44, 60, 119). These studies indicated that employees’ high degree of confidence in the efficacy of security measures enhanced their cybersecurity behavior during digital transformation. In other words, when employees have a clear perception that their preventive measures are effective, they tend to adhere to better cybersecurity practices.

## 7 Contributions and future work

### 7.1 Theoretical contributions

This paper’s findings provide several theoretical contributions to the field of sector.

The study contributes by proposing a new research model that combines two fundamental theories: general deterrence theory (GDT) and protection motivation theory (PMT).

Firstly, GDT was used to examine the impact of certainty of punishment on trust and on reducing vulnerability to exploitation. This theory significantly contributed to the model and improved the study’s results. The theory explained that certainty of punishment reduces vulnerability to misuse of digital systems, thereby increasing employee trust in digital technologies during the digital transformation process.

Secondly, through applying PMT, threats associated with digital transformation, such as privacy violations and technological complexity, were identified as threats that motivate employees to protect the digital environment and enhance their cybersecurity behavior within healthcare organizations.

This study’s third theoretical contribution is its focus on both human and technical factors and how they influence cybersecurity during digital transformation. The study is one of the first to examine the direct impact of the cyber-threats associated with digital transformation, such as exploitability and complexity, on employee cybersecurity compliance behavior, enriching theoretical understanding of the factors influencing cybersecurity within digital workplaces.

In addition, the most important behavioral factors (i.e., privacy, trust, and security awareness) were examined as precursors to employee cybersecurity behavior in healthcare settings. Furthermore, the study used a two-stage PLS-SEM–ANN analysis to investigate the factors that significantly influenced employee cybersecurity compliance during digital transformation. By combining the best features of both approaches, the hybrid approach improved the results’ accuracy (92). Furthermore, the study makes recommendations for further research combining machine learning (ML) and structural equation modeling (SEM) methods (39, 91, 108).

The results of the dual analysis were consistent in terms of the relative importance of each factor. Consequently, this study contributes to knowledge by directly assessing the relative importance of these factors, demonstrating their conceptual and practical significance.

### 7.2 Practical implications

The study offers several important practical implications for healthcare organizations during digital transformation.

Firstly, it provides a deeper understanding of the factors influencing cybersecurity compliance behavior. This understanding helps organizations not only to design more effective cybersecurity measures based on employee behaviors and attitudes, but also to develop strategies and policies that enhance employee cybersecurity compliance and mitigate cyber risks.

Secondly, the study reveals the importance of cybersecurity awareness, which, based on the dual analysis results, ranked first, followed by trust in digital systems as key factors influencing employee cybersecurity compliance. Accordingly, healthcare organizations can develop customized training programs that focus on these factors to raise cybersecurity awareness among employees (39, 116).

Thirdly, this study was not limited to examining employee cybersecurity behavior toward a specific technology, unlike some previous studies that focused on specific forms of technology, such as electronic health records (EHRs) (92) and biometrics continuous authentication (BBCA) (56). This enhances the reliability and applicability of the findings across multiple technical contexts to develop more workplace-friendly cybersecurity technology systems.

Previously, employee cybersecurity behavior surveys were conducted in various countries, such as Slovenia (89), Jordan (92), the United States (US) (62), and Malaysia (60). Differences in culture, infrastructure, legislation, and national economy may influence the decision-making process in developing cybersecurity strategies and policies (92).

Therefore, this is the first empirical study responding to calls for action from a Saudi Arabian perspective. It was not limited to a specific healthcare facility or city in Saudi Arabia, as was the case in Arar city (15). Therefore, the study’s findings contribute to the work of decision-makers who are developing more comprehensive cybersecurity strategies and policies applicable to various Saudi Arabian healthcare facilities that are undergoing digital transformation.

### 7.3 Social contributions

One of the most significant social contributions of this research is to raise cybersecurity awareness among employees, as they become more aware of cyber risks, threats, and appropriate protection methods. This reduces the likelihood of making mistakes that could lead to the leakage of sensitive data (120).

Furthermore, the research contributes to employees’ awareness of the need for privacy in the use of digital systems. It makes them more aware of the importance of maintaining their privacy when using digital technologies, such as using strong passwords, regularly updating systems, and activating anti-malware and anti-virus systems.

The research also helps to reduce the psychological and professional stress that employees may experience due to fears associated with digital system breaches, creating a more stable and secure work environment.

Finally, the social contribution of this research is not limited to healthcare workers but extends to all individuals across various sectors. By fostering a safe work environment, reducing cyber risks, and improving employee efficiency, the research contributes to promoting the safer, more reliable, and higher-quality use of technologies, thus supporting the success of digital transformation in the Kingdom of Saudi Arabia (KSA).

### 7.4 Research limitations and future work

Despite the study’s valuable findings, some limitations are identified that provide opportunities for future research to deepen understanding and expand the study’s scope.

Firstly, as the sample was limited to healthcare sector employees from the Kingdom of Saudi Arabia (KSA), generalizing the results to other countries may be challenging due to differences in policies and organizational culture regarding cybersecurity. Therefore, future research could be conducted across different countries.

Secondly, one of the study’s methodological limitations is the use of the snowball sampling method to recruit participants, relying on participant nominations to attract more participants. While this method is useful for reaching employees in environments where data collection is difficult (83), it may lead to sample bias, as participants may be limited to certain employee categories and not represent all healthcare professions. Therefore, future research could use other data collection techniques.

Furthermore, Alhuwail et al. (116) indicate that job experience plays a role in influencing compliance with cybersecurity measures, as older or more experienced employees may be more committed to cybersecurity measures than novices. However, as this was not among the study’s objectives, this aspect was not addressed, so no comparisons were made with regard to employees’ years of experience. Therefore, future research could examine the impact of recognized years of experience on employee cybersecurity behaviors.

Finally, the proposed model has not addressed some factors associated with digital transformation that may have an impact, such as ease of use and availability. Therefore, future research could consider these factors and examine how they influence improved cybersecurity behavior.

## 8 Conclusion

Amid the acceleration of digital transformation, healthcare has been experiencing a rise in hacking and security breaches, prompting the need for this study. By integrating general deterrence theory (GDT) and protection motivation theory (PMT), this study developed a research model for gaining an understanding of the key factors and examined the relationship between these theories and their impact on employee cybersecurity behavior during digital transformation. The research model was developed and empirically tested using PLS-ANN analysis, with data collected from 252 participants working in the healthcare sector.

The study’s most prominent findings were that certainty of punishment had a significant impact on trust as well as reducing vulnerability to exploitation.

Privacy and system complexity were shown to increase perceived threat and vulnerability, influencing protective motivations. Notably, most Protection Motivation Theory (PMT) factors did not directly affect cybersecurity behavior, except for response efficacy, which reflects employees’ confidence in the digital and security systems used.

Furthermore, the paper revealed that cybersecurity awareness and trust have direct effects on employees’ cybersecurity behaviors in the healthcare sector. Cybersecurity awareness plays a crucial role in helping employees recognize potential threats and take proactive steps to protect sensitive healthcare information.

The conclusion also highlighted the study’s theoretical, practical, and social contributions, along with the challenges it faced and its recommendations for future research. Ultimately, the study successfully achieved its research objectives and answered its research questions.

## Data Availability

The raw data supporting the conclusions of this article will be made available by the authors, without undue reservation.

## Footnotes

^1^The abbreviation ‘EU’ indicates that this is a regulation of the European Parliament and the Council of the European Union.
