# Supplementary material for: Cybersecurity governance in the healthcare sector during digital transformation: an integrated model and hybrid analytical approach
Source: Front Public Health. 2025 Nov 19;13:1703689. doi: 10.3389/fpubh.2025.1703689 (PMC12672465; doi:10.3389/fpubh.2025.1703689)
Supplement: Supplementary file 1 [file Table_1.pdf]

Appendix A :

Table : Survey Instruments

Construct	Code	Items	Adapted from
Privacy	*PRV1*	I am concerned that my personal information may be used for wrong purposes.	Skalkos et al. 2021
	PRV2	I am concerned that my health information is being used for wrong purposes.	
	PRV3	I feel that my personal information is adequately protected in the hospital.	
	PRV4	I believe that the use of technology in healthcare may violate my privacy.	
Perceived severity	*PS1*	The vulnerability of digital systems in healthcare to breaches is a serious problem for me.	Li et al. 2022
	PS2	Anyone having access to confidential information is a serious problem for me.	
	PS3	I think the increase in digital systems in healthcare is a threat to cybersecurity.	
	PS4	I believe that the lack of awareness about digital systems and their risks is a threat to me.	
Perceived vulnerability	*PV1*	I feel that my health organization may be vulnerable to security breaches due to digital systems and devices.	Li et al. 2022
	PV2	I believe that the data I am dealing with may be targeted by cyber attackers.	
	PV3	I am concerned that the technology systems I use are not adequately protected against cyber-attacks.	
Self-efficacy	*SE1*	I have the competencies to implement security measures, such as using a strong password.	Alanazi et al. 2020
	SE2	Taking the necessary security measures is easy.	Kimpe et al. 2022
	SE3	I believe I have the skills to effectively use digital technology in healthcare.	-
	SE4	I feel I can effectively protect the privacy of health data.	-
Response efficacy	*RE1*	Adhering to my organization's security policies will reduce the likelihood of security breaches.	Li et al. 2022
	RE2	My health organization's security measures are effective.	-
	RE3	I believe that using certified security software greatly helps in protecting health data.	-

Construct	Code	Items	Adapted from
Trust	*T1*	I trust digital systems in healthcare.	Skalkos et al. 2021
	T2	I feel good when using digital systems as they are generally reliable and accurate.	
	T3	Developing policies on the use of digital health systems increases trust.	
	T4	I feel that my cybersecurity behavior is influenced by the level of trust I feel towards my organization's security systems.	
Certainty of punishment	*CP1*	I believe that misuse of technology can usually be corrected with punishment.	Alanazi et al. 2020
	CP2	I believe that imposing a definite penalty on those who violate the protection of digital systems will increase confidence in technology.	-
	CP3	I believe that imposing a specific penalty reduces the exploitation of vulnerabilities in digital systems.	-
Complexity	*C1*	The use of multiple digital systems and devices increases the complexity of healthcare.	-
	C2	I hesitate to adopt modern technologies due to their complexity.	Zhan et al. 2024
	C3	I avoid using health information systems due to their complexity.	
	C4	The complexity of technology in health systems increases the potential for cyber threats.	-
Awareness	*AW1*	I have a sufficient understanding of the threats that digital technologies currently pose to my health organization.	Garcia-Perez et al. 2023
	AW2	Current digital systems have increased my organization's cybersecurity risks.	
	AW3	I follow news and developments about security-related digital technologies.	Alanazi et al. 2020
	AW4	I have a good understanding of the digital systems in my health organization.	
Exploitability	*EX1*	I feel that not strictly enforcing security policies increases the risk of exploitation.	-
	EX2	I feel that the complexity of health systems increases the vulnerability to exploitation.	
	EX3	I feel that exploitability affects the adoption of digital technologies in healthcare.	
Cybersecurity behavior during digital transformation	*CDT1*	Security updates are applied regularly to my organization's systems.	-
	CDT2	My organization has an effective plan for keeping health data secure.	Garcia-Perez et al. 2023
	CDT3	I adhere to all cybersecurity procedures put in place by my health organization.	-

Construct	Code	Items	Adapted from
	CDT4	I monitor unusual computer behavior or responses (e.g., computer slowdowns or freezes, pop-ups, etc.).	Li et al. 2022
	CDT5	I feel that employing digital transformation in my health organization enhances cybersecurity.	
